# Gut microbiota and postpartum depression: a Mendelian randomization study

**DOI:** 10.3389/fpsyt.2024.1282742

**Published:** 2024-06-03

**Authors:** Jianjun Zhang, Lechuan Wei, Hongfei Tan, Wenwen Pang

**Affiliations:** ^1^ Department of Obstetrics, Affiliated Hospital of Weifang Medical University, Weifang, China; ^2^ Weifang Key Laboratory of Postpartum Pelvic Floor electromyography (EMG) Rehabilitation, Weifang, China; ^3^ Clinical College of Weifang Medical University, Weifang, China

**Keywords:** Mendelian randomization, gut microbiota, postpartum depression (PPD), causal relationship, w-3

## Abstract

**Background:**

Increasing evidence suggests a correlation between intestinal microbiota and the gut-brain axis; however, the causal relationship between gut microbiota and postpartum depression (PPD) remains unclear.

**Methods:**

In this study, a two-sample Mendelian randomization (MR) design was employed to analyze the GWAS data of gut microorganisms from the Mibiogen database and PPD data from the UK biobank. Various statistical methods, including inverse variance weighted, MR-Egger, weighted median, weighted model, and MR-PRESSO, were utilized to investigate the causal relationship between gut microbiota and PPD. Additionally, sensitivity analysis was conducted to assess the robustness of the findings.

**Results:**

Through MR analysis, it was found that phylum Actinobacteria (*P*=0.014, OR=0.971, 95% CI=0.948-0.994) and genus Holdemanella (*P*=0.023, OR=0.979, 95% CI=0.961-0.997) have protective effects on PPD, while the other two unknown genera, genus Unknown Ids 2001 (*P*=0.025, OR=0.972,95% CI=0.947-0.996), and genus Unknown Ids 2755 (*P*=0.012, OR=0.977, 95% CI=0.959-0.995) also has a protective effect on PPD. The sensitivity analysis results indicate that there is no heterogeneity or horizontal pleiotropy.

**Conclusion:**

This study has identified a causal association between Actinomycetota, Holdemanella, and PDD through MR analysis. These findings offer significant contributions to the development of personalized treatment approaches for PPD, encompassing interventions such as dietary modifications or microbiome interventions.

## Introduction

Postpartum depression (PPD) is a non-psychotic depressive episode that begins or continues into the postpartum period (1). Postpartum depression is the most common complication of childbirth, affecting women and mother-infant relationships and cognitive and emotional problems in children, with serious consequences for mothers, families, and children (2). Nearly 20% of patients with depression relapse within 20 years of their initial onset, and most people develop suicidal thoughts, with 4% -5% dying from depression-related suicide (3, 4). Not only does it significantly impact the mother itself, but it also affects the quality of life of a family.

Recent studies have shown that the gut microbiota (GM) plays an important physiological role in maintaining gastrointestinal, hormonal, immune, and neural homeostasis (5). The concept of the “microbiota-gut-brain (MGB) axis” has been developed to understand the impact of the gut-brain axis on human homeostasis, particularly in the field of psychiatry (6). There is a close relationship between depression and the microbiota, as recent research suggests that the gut microbiota may have a significant impact on the onset and development of depression. Animal experiments indicate that the gut microbiota can influence brain function and potentially affect behavior. For example, oral administration of Lactobacillus can reduce the expression of pro-inflammatory cytokines and increase the levels of BDNF in the hippocampus, leading to anti-anxiety and antidepressant effects in mice (5).

Evidence from human studies indicates that the gut microbiota of individuals with depression differs significantly from that of healthy individuals, including changes in the abundance of specific bacterial genera and alterations in the overall microbial community structure. Some microbial families have been found to be positively associated with anxiety and depressive symptoms, while others may help alleviate depressive symptoms (7–9). Additionally, the relative abundance of certain bacterial taxa, such as the Firmicutes phylum, appears to be more representative in major depressive disorder (MDD) (6, 10). This association is likely mediated through mechanisms such as regulating inflammation, influencing neurotransmitter synthesis and metabolism, and modulating the gut-brain axis signaling.Overall, the research suggests that the gut microbiota may have a profound impact on the molecular pathways involved in the occurrence and development of anxiety and depression-related behaviors, despite the differences between human and murine microbiomes (11).

Mendelian randomization (MR) is a new approach to exploring the causal relationship between gut microbiota and PPD by constructing working exposure variables using genetic variation to assess the causal relationship between exposure and outcome (12). Due to the random assignment of genes, the influence of other confounding factors is also avoided (13). The Mibiogen database is a bioinformatics platform that can be used for multi-omics data analysis and interactive visualization (14), based on which numerous authors have explored the causal relationship between gut flora and a variety of diseases, including eclampsia (15), adverse pregnancy outcomes (16) and ischemic Stroke (17).

In this study, a two-sample MR Analysis was performed using pooled statistics from genome-wide Association Studies (GWAS) from MiBioGen and the UK biobank consortium to explore specific gut microbiota causally associated with PPD.

## Methods

### Data sources

We obtained summary statistics of genome-wide association studies of the gut microbiota in mibiogen (18), and the MiBioGen study coordinated 2021S rRNA gene sequencing profiles and genome-wide genotyping data from 18,473 individuals (25 cohorts) and is the largest, multi-ethnic, genome-wide meta-analysis of the gut microbiome to date (19). This study included 211 taxa: 9 phylum, 16 orders, 20 families, 35 families, 131 genera, and 7738 participants of European ancestry, as determined by 16S ribosomal RNA gene sequencing (18). Data for PPD were obtained from the UK biobank, containing 4834 patients and 33173 controls from the European population, containing a total of 11,982,120 SNPs (20). All the people are European.

### Genetic variants selection criteria

Based on the screening criteria from previous literature, we chose a stringent threshold of P<1×10^-5^ to select instrumental variables (IVs) for our analysis. This threshold ensures that only genetic variants with a very low probability of being associated with the outcome are included as IVs, reducing the likelihood of including SNPs with weak or spurious associations.

Additionally, to ensure the independence of each IV, we applied a threshold of r^2^<0.001 within a window size of 10,000 kb. This step aimed to mitigate the effects of linkage disequilibrium (LD), a phenomenon where genetic variants close to each other on the chromosome are inherited together. By trimming IVs that are in high LD with each other, we aimed to reduce redundancy and remove SNPs that are essentially providing the same information. This helps in ensuring that the selected IVs are truly independent and provide unique information for the analysis.

Furthermore, we removed “echo SNPs” which are SNPs that are redundant due to LD and do not provide additional information beyond the already included SNPs. We also excluded SNPs that were not present in the results from the IVs, ensuring that all SNPs used in the analysis had valid and reliable data available for the research.

By applying these stringent criteria, we aimed to ensure that the selected IVs were robust, independent, and unlikely to be influenced by LD, thus enhancing the quality and reliability of our instrumental variable analysis.

### MR analysis

The IVW method is an extension of the Wald ratio estimator based on meta-analysis principles (21). The random effects model with inverse variance weight was selected as the main MR method. For the flora with causality in IVW (p<0.05), four additional methods were selected as supplements (MR Egger, weighted median, simple model, and Weighting pattern). In addition, we conducted a sensitivity analysis of the results. Firstly, we used the MR Egger interception test and the MR PRESSO global test to detect horizontal pleiotropy (22, 23). We reported the heterogeneity of the Wald estimator using the Cochrane Q statistic (24). In addition, a retention analysis was conducted to evaluate the robustness of the results.

All analyses in this study were conducted based on R software (version 4.2.1). The “TwoSampleMR” R package and the “MRPRESSO” R package were used for our MR research.

## Results

According to the selection criteria of IVs, a total of 2044 SNPs were used as IVs for 5 levels and 211 sets, including 9 phylum, 16 classes, 20 orders, 35 families, and 119 bacterial genera.

**Figure 1:**
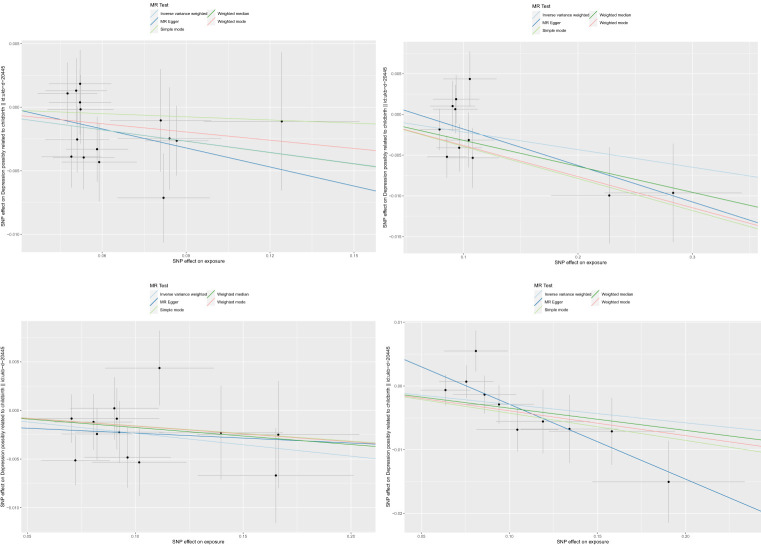
Scatter plots for the causal association between gut microbiota and PPD.

We tested the causal relationship between Gut microbiota and postpartum depression by five MR methods. We identified a causal relationship between four bacterial characteristics and postpartum depression using the IVW method (**Table 1**, **Figure 1**). They are phylum Actinobacteria (P=0.014, OR=0.971,95% CI=0.948-0.994), genus Holdemanella (P=0.023, OR=0.979,95% CI=0.961-0.997), genus. unknown. ids. 2001 (P=0.025, OR=0.972,95% CI=0.947-0.996), and genus. unknown. ids. 2755 (P=0.012, OR=0.977,95% CI=0.959-0.995). They contain 15, 11, 10, and 13 SNPs, respectively. Additionally, other methods were used to compare The screened strains were validated, and beta values in the same direction were also obtained, Proving that our results are robust.

**Table 1:** Causal estimations of gut microbiota on postpartum depression in the MR analysis.

exposure	method	nsnp	b	pval	OR	95%CI
**Phylum** Actinobacteria id.400	MR Egger	15	-0.050	0.314	0.951	(0.866~1.044)
	Weighted median		-0.030	0.065	0.971	(0.941~1.002)
	IVW		-0.030	0.014	0.971	(0.948~0.994)
	Simple mode		-0.008	0.781	0.922	(0.936~1.051)
	Weighted mode		-0.022	0.435	0.979	(0.928~1.032)
**Genus** Holdemanella id.11393	MR Egger	11	-0.045	0.139	0.956	(0.906~1.009)
	Weighted median		-0.032	0.010	0.969	(0.945~0.992)
	IVW		-0.022	0.023	0.979	(0.961~0.997)
	Simple mode		-0.039	0.108	0.961	(0.92~1.004)
	Weighted mode		-0.038	0.072	0.963	(0.927~0.999)
**Genus** Unknowngens id.2755	MR Egger	13	-0.010	0.792	0.990	(0.919~1.066)
	Weighted median		-0.018	0.160	0.983	(0.959~1.007)
	IVW		-0.023	0.012	0.977	(0.959~0.995)
	Simple mode		-0.016	0.451	0.984	(0.946~1.024)
	Weighted mode		-0.016	0.433	0.984	(0.947~1.023)
**Genus** unknowngenus id.2001	MR Egger	10	-0.117	0.015	0.889	(0.825~0.958)
	Weighted median		-0.035	0.034	0.966	(0.935~0.997)
	IVW		-0.029	0.025	0.972	(0.947~0.996)
	Simple mode		-0.043	0.105	0.958	(0.915~1.004)
	Weighted mode		-0.039	0.153	0.962	(0.916~1.01)

IVW, inverse variance weighted; MR, Mendelian randomization; nsnp, number of single‐nucleotide polymorphism;b,bata; OR, odds ratio; SM, Simple mode; 95%CI, 95% Confidence interval.

**Figure 2:**
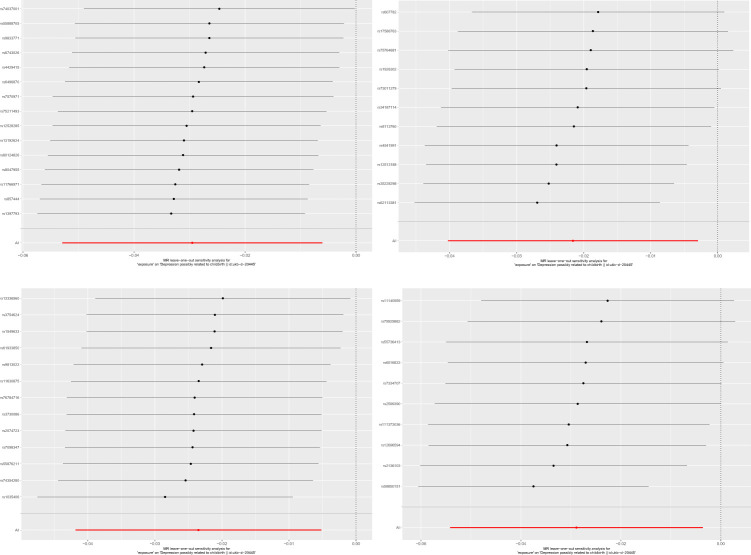
Leave-one-out analysis for the causal association between gut microbiota and PPD.

We used IVW testing and MR Egger regression to test the Q-statistic results and did not find any heterogeneity in the results. We used MR Egger regression to detect the presence of horizontal pleiotropy in Genus (**Table 2**). Holdemanella (P=0.04), but no other results showed the presence of horizontal pleiotropy. At the same time, we used the MR-PRESSO algorithm for detection and did not find the existence of horizontal pleiotropy. The forest diagram of causal effects using a single SNP shows that their association with mental illness/traits is not very significant, and sensitivity analysis indicates that there is no single SNP driving causal association signal (**Figure 2**).

**Table 2:** Heterogeneity test and horizontal pleiotropy test of gut microbiota on postpartum depression.

exposure¤	method¤	Heterogeneity test¤			horizontal pleiotropy test¤		MR PRESSO¤
		Q¤	Q_df¤	Q_pval¤	egger¶ intercept¤	pval¤	
**Phylum¶** Actinobacteria¶ id.400¤	MR Egger¤	9.817¤	13¤	0.709¤	0.001¤	0.665¤	0.788¤
	IVW¤	10.013¤	14¤	0.761¤	¤	¤	
**Genus¶** Holdemanella¶ id.11393¤	MR Egger¤	5.851¤	8¤	0.664¤	0.009¤	0.041¤	0.368¤
	IVW¤	11.792¤	9¤	0.225¤	¤	¤	
**Genus¶** Unknowngens¶ id.2755¤	MR Egger¤	7.773¤	11¤	0.733¤	-0.001¤	0.722¤	0.817¤
	IVW¤	7.907¤	12¤	0.792¤	¤	¤	
**Genus¶** Unknowngens¶ id.2001¤	MR Egger¤	10.343¤	9¤	0.323¤	0.003¤	0.395¤	0.258¤
	IVW¤	11.262¤	10¤	0.337¤	¤	¤	

p value > 0.05 represent no significant pleiotropy. Q_p value > 0.05 represents no significant heterogeneity.

GWAS, genome‐wide association study; IVs, instrumental variants; IVW, inverse variance weighted; MR, Mendelian randomization; SE, standard error.

In addition, the MR Steiger directionality test showed that the variance explained by the included bacterial exposure SNP was greater than the mental outcome, indicating a true causal correlation in the direction.

## Discussion

In this study, the causal relationship between four bacterial features in the gut microbiota genome-wide association study (GWAS) and postpartum depression (PPD) was demonstrated through Mendelian randomization (MR) analysis. This research is not only significant in understanding the role of the gut microbiota in postpartum depression, but also provides new evidence for the “microbiota-gut-brain (MGB) axis” concept. The gut-brain axis is involved in the shared genetic basis of gastrointestinal and mental disorders, a notion which has been confirmed through comprehensive genomic range analysis (25).

This study identified a causal relationship between four bacterial genera and postpartum depression (PPD), allowing for in-depth exploration of the impact of these microbial changes on function and metabolism. Notably, the research on Actinobacteria and Holdemanella genera is particularly intriguing. Evidence of the protective role of Actinobacteria as a key member of the gut microbiota against depression continues to accumulate, as demonstrated by Tian et al.’s analysis of the gut microbiota in normal mice and those with PPD, which revealed higher abundance of Actinobacteria (including Bifidobacterium and Corynebacterium) in the normal group compared to the PPD group (26). Previous research has also indicated an association between gut microbiota imbalance and certain mental disorders such as anxiety and depression. Jiang’s high-throughput sequencing analysis of 46 depressed patients and 30 healthy controls showed significantly higher abundance of Actinobacteria and Firmicutes in the healthy control group at the phylum level compared to the depressed patients (27). Moreover, previous MR studies have indicated a protective effect of Actinobacteria against major depressive disorder (MDD) incidence (OR 0.88, 95% CI 0.87-0.9) (28). Therefore, bacteria within the Actinobacteria phylum may indirectly influence the onset and development of mental disorders by affecting the balance of the gut microbiota. In fact, Actinobacteria are producers of many important antibiotics (29), including penicillin, tetracycline, and erythromycin, and their increased abundance may compete with pathogens for nutrients and adhesion sites, thereby inhibiting pathogen colonization and growth, and contributing to the maintenance of gut microbiota balance. Additionally, some Actinobacteria may modulate the host’s immune system, contributing to immune response regulation and maintaining gut immune system balance. However, further research and exploration are needed to elucidate the specific mechanisms and effects of Actinobacteria in alleviating postpartum depression.

Research on the Holdemanella genus also suggests its potential beneficial impact in reducing the risk of postpartum depression (PPD). In a study on post-stroke depression (PSD), researchers analyzed fecal samples from 232 patients with acute ischemic stroke using 16S rRNA sequencing. The samples were assessed using the Hamilton Depression Rating Scale (HAMD-3). The results indicated a significant decrease in the abundance of Holdemanella genus in PSD patients, and a negative correlation between the abundance of Holdemanella genus and HAMD scores, suggesting a potential beneficial impact of Holdemanella genus in reducing the risk of PSD (30). Furthermore, Jiang’s study observed lower abundance of Firmicutes in the gut microbiota of depressed patients compared to healthy controls (7). Additionally, several studies consistently indicate that increasing the levels of the Holdemanella genus is beneficial in reducing the incidence of depression (31, 32). It is worth mentioning that depressed patients often have lower levels of omega-3 fatty acids (33). While there is no consensus on whether supplementing omega-3 alone can effectively alleviate depression, it has been observed that consuming omega-3-rich fish may be associated with increased abundance of Holdemanella genus (27). Could increasing the abundance of Holdemanella genus and reducing the risk of postpartum depression be achieved through omega-3 supplementation? This is purely speculative, but it also provides a new perspective on the role of dietary intervention in preventing postpartum depression.

This study identified a causal relationship between four bacterial features in the gut microbiota genome-wide association study (GWAS) and postpartum depression (PPD) through Mendelian randomization (MR) analysis. Additionally, it suggests that the Actinobacteria and Holdemanella genera may have a potential beneficial impact in reducing the risk of depression. Adjusting the abundance of these microorganisms in the gut microbiota may help improve symptoms of certain mental disorders, providing important evidence for understanding the role of the gut microbiota in postpartum depression.

However, this study also has some limitations. Firstly, the GWAS meta-analysis of the gut microbiota included male and female participants. Even though genetic variants located on the sex chromosomes were excluded from the analysis and adjustments for gender were made, it may still introduce bias (18). Moreover, the majority of the data is from individuals of European descent, potentially introducing interference from racial differences. Therefore, in future studies, we hope to conduct detailed subgroup analyses targeting specific populations to explore the influence of gender on the relationship between the gut microbiota and PDD. Additionally, we aim to conduct in-depth research on specific gut microbiota to understand their association with PDD, and further explore the mechanisms of specific microbiota in PDD through metagenomic analysis and functional experiments, deepening our understanding of the relationship between the gut microbiota and PDD, and providing a scientific basis for more precise intervention measures in the future.

## Data availability statement

The datasets presented in this study can be found in online repositories. The names of the repository/repositories and accession number(s) can be found in the article/supplementary material.

## Author contributions

JZ: Conceptualization, Data curation, Formal analysis, Methodology, Project administration, Supervision, Writing – original draft, Writing – review & editing. LW: Conceptualization, Data curation, Formal analysis, Writing – original draft. HT: Data curation, Formal analysis, Validation, Writing – original draft. WP: Conceptualization, Data curation, Formal analysis, Methodology, Project administration, Supervision, Validation, Writing – original draft, Writing – review & editing.

## References

[1] Righetti-Veltema M Conne-Perréard E Bousquet A Manzano J. Risk factors and predictive signs of postpartum depression. J Affect Disord (1998) 49(3):167–80. doi: 10.1016/S0165-0327(97)00110-9 9629946

[2] Stein A Pearson RM Goodman SH Rapa E Rahman A McCallum M. Effects of perinatal mental disorders on the fetus and child. Lancet (2014) 384(9956):1800–19. doi: 10.1016/S0140-6736(14)61277-0 25455250

[3] Sim K Lau WK Sim J Sum MY Baldessarini RJ. Prevention of relapse and recurrence in adults with major depressive disorder: systematic review and meta-analyses of controlled trials. Int J Neuropsychopharmacol (2015) 19(2). doi: 10.1093/ijnp/pyv076 PMC4772815 26152228

[4] Miret M Ayuso-Mateos JL Sanchez-Moreno J Vieta E. Depressive disorders and suicide: Epidemiology, risk factors, and burden. Neurosci Biobehav Rev (2013) 37(10 Pt 1):2372–4. doi: 10.1016/j.neubiorev.2013.01.008 23313644

[5] Guo Y Xie JP Deng K Li X Yuan Y Xuan Q. Prophylactic effects of bifidobacterium adolescentis on anxiety and depression-like phenotypes after chronic stress: A role of the gut microbiota-inflammation axis. Front Behav Neurosci (2019) 13:126. doi: 10.3389/fnbeh.2019.00126 31275120 PMC6591489

[6] Chen JJ Zheng P Liu YY Zhong XG Wang HY Guo YJ. Sex differences in gut microbiota in patients with major depressive disorder. Neuropsychiatr Dis Treat (2018) 14:647–55. doi: 10.2147/NDT.S159322 PMC5833751 29520144

[7] Jiang H Ling Z Zhang Y Mao H Ma Z Yin Y. Altered fecal microbiota composition in patients with major depressive disorder. Brain Behav Immun (2015) 48:186–94. doi: 10.1016/j.bbi.2015.03.016 25882912

[8] Slykerman RF Hood F Wickens K Thompson JMD Barthow C Murphy R. Effect of lactobacillus rhamnosus HN001 in pregnancy on postpartum symptoms of depression and anxiety: A randomised double-blind placebo-controlled trial. EBioMedicine (2017) 24:159–65. doi: 10.1016/j.ebiom.2017.09.013 PMC5652021 28943228

[9] Zheng P Zeng B Zhou C Liu M Fang Z Xu X. Gut microbiome remodeling induces depressive-like behaviors through a pathway mediated by the host's metabolism. Mol Psychiatry (2016) 21(6):786–96. doi: 10.1038/mp.2016.44 27067014

[10] Lin P Ding B Feng C Yin S Zhang T Qi X. Prevotella and Klebsiella proportions in fecal microbial communities are potential characteristic parameters for patients with major depressive disorder. J Affect Disord (2017) 207:300–4. doi: 10.1016/j.jad.2016.09.051 27741466

[11] Bibbò S Fusco S Ianiro G Settanni CR Ferrarese D Grassi C. Gut microbiota in anxiety and depression: Pathogenesis and therapeutics. Front Gastroenterol (2022) 1:1019578. doi: 10.3389/fgstr.2022.1019578 PMC12952365 41822071

[12] Greenland S. An introduction to instrumental variables for epidemiologists. Int J Epidemiol (2000) 29(4):722–9. doi: 10.1093/ije/29.4.722 10922351

[13] Burgess S Thompson SG. Multivariable Mendelian randomization: the use of pleiotropic genetic variants to estimate causal effects. Am J Epidemiol (2015) 181(4):251–60. doi: 10.1093/aje/kwu283 PMC4325677 25632051

[14] Swertz MA Dijkstra M Adamusiak T van der Velde JK Kanterakis A Roos ET. The MOLGENIS toolkit: rapid prototyping of biosoftware at the push of a button. BMC Bioinf (2010) 11 Suppl 12(Suppl 12):S12. doi: 10.1186/1471-2105-11-S12-S12 PMC3040526 21210979

[15] Li P Wang H Guo L Gou X Chen G Lin D. Association between gut microbiota and preeclampsia-eclampsia: a two-sample Mendelian randomization study. BMC Med (2022) 20(1):443. doi: 10.1186/s12916-022-02657-x 36380372 PMC9667679

[16] Li C Liu C Li N. Causal associations between gut microbiota and adverse pregnancy outcomes: A two-sample Mendelian randomization study. Front Microbiol (2022) 13:1059281. doi: 10.3389/fmicb.2022.1059281 36590417 PMC9801412

[17] Meng C Deng P Miao R Tang H Li Y Wang J. Gut microbiome and risk of ischaemic stroke: a comprehensive Mendelian randomization study. Eur J Prev Cardiol (2023) 30(7):613–20. doi: 10.1093/eurjpc/zwad052 36799937

[18] Kurilshikov A Medina-Gomez C Bacigalupe R Radjabzadeh D Wang J Demirkan A. Large-scale association analyses identify host factors influencing human gut microbiome composition. Nat Genet (2021) 53(2):156–65. doi: 10.1038/s41588-020-00763-1 PMC8515199 33462485

[19] Wang J Kurilshikov A Radjabzadeh D Turpin W Croitoru K Bonder MJ. Meta-analysis of human genome-microbiome association studies: the MiBioGen consortium initiative. Microbiome (2018) 6(1):101. doi: 10.1186/s40168-018-0479-3 29880062 PMC5992867

[20] Hemani G Zheng J Elsworth B Wade KH Haberland V Baird D. The MR-Base platform supports systematic causal inference across the human phenome. Elife (2018) 7. doi: 10.7554/eLife.34408 PMC5976434 29846171

[21] Pagoni P Dimou NL Murphy N Stergiakouli E. Using Mendelian randomisation to assess causality in observational studies. Evid Based Ment Health (2019) 22(2):67–71. doi: 10.1136/ebmental-2019-300085 30979719 PMC10270458

[22] Rees JMB Wood AM Burgess S. Extending the MR-Egger method for multivariable Mendelian randomization to correct for both measured and unmeasured pleiotropy. Stat Med (2017) 36(29):4705–18. doi: 10.1002/sim.7492 PMC5725762 28960498

[23] Verbanck M Chen CY Neale B Do R. Detection of widespread horizontal pleiotropy in causal relationships inferred from Mendelian randomization between complex traits and diseases. Nat Genet (2018) 50(5):693–8. doi: 10.1038/s41588-018-0099-7 PMC6083837 29686387

[24] Bowden J Del Greco MF Minelli C Davey Smith G Sheehan N Thompson J. A framework for the investigation of pleiotropy in two-sample summary data Mendelian randomization. Stat Med (2017) 36(11):1783–802. doi: 10.1002/sim.7221 PMC5434863 28114746

[25] Gong W Guo P Li Y Liu L Yan R Liu S. Role of the gut-brain axis in the shared genetic etiology between gastrointestinal tract diseases and psychiatric disorders: A genome-wide pleiotropic analysis. JAMA Psychiatry (2023) 80(4):360–70. doi: 10.1001/jamapsychiatry.2022.4974 PMC9909581 36753304

[26] Tian XY Xing JW Zheng QQ Gao PF. 919 syrup alleviates postpartum depression by modulating the structure and metabolism of gut microbes and affecting the function of the hippocampal GABA/glutamate system. Front Cell Infect Microbiol (2021) 11:694443. doi: 10.3389/fcimb.2021.694443 34490139 PMC8417790

[27] Jiang CH Fang X Huang W Guo JY Chen JY Wu HY. Alterations in the gut microbiota and metabolomics of seafarers after a six-month sea voyage. Microbiol Spectr (2022) 10(5):e0189922. doi: 10.1128/spectrum.01899-22 36197290 PMC9603232

[28] Chen M Xie CR Shi YZ Tang TC Zheng H. Gut microbiota and major depressive disorder: A bidirectional Mendelian randomization. J Affect Disord (2022) 316:187–93. doi: 10.1016/j.jad.2022.08.012 35961601

[29] Barka EA Vatsa P Sanchez L Gaveau-Vaillant N Jacquard C Meier-Kolthoff JP. Taxonomy, physiology, and natural products of actinobacteria. Microbiol Mol Biol Rev (2016) 80(1):1–43. doi: 10.1128/MMBR.00019-15 26609051 PMC4711186

[30] Yao S Xie H Wang Y Shen N Chen Q Zhao Y. Predictive microbial feature analysis in patients with depression after acute ischemic stroke. Front Aging Neurosci (2023) 15:1116065. doi: 10.3389/fnagi.2023.1116065 37032826 PMC10076592

[31] Chen Y Meng P Cheng S Jia Y Wen Y Yang X. Assessing the effect of interaction between C-reactive protein and gut microbiome on the risks of anxiety and depression. Mol Brain (2021) 14(1):133. doi: 10.1186/s13041-021-00843-1 34481527 PMC8418706

[32] Zhang H Liu L Cheng S Jia Y Wen Y Yang X. Assessing the joint effects of brain aging and gut microbiota on the risks of psychiatric disorders. Brain Imaging Behav (2022) 16(4):1504–15. doi: 10.1007/s11682-022-00630-z 35076893

[33] Lin PY Huang SY Su KP. A meta-analytic review of polyunsaturated fatty acid compositions in patients with depression. Biol Psychiatry (2010) 68(2):140–7. doi: 10.1016/j.biopsych.2010.03.018 20452573

